# Erratum

**DOI:** 10.1080/13102818.2014.978176

**Published:** 2014-11-14

Petranova, T., Sheytanov, I., Monov, S., Rashkov, R. and Kinov, P. (2013). Efficacy of Denosumab for Control of Bone Mineral Density and Microarchitecture in Postmenopausal Women with Osteoporosis: A One-Year Experience. *Biotechnology & Biotechnological Equipment*, 27(4), pp. 3977–3981. http://dx.doi.org/10.5504/BBEQ.2013.0045

When the above article was first published online, there was a discrepancy between the figures and the figure captions. This is now corrected as follows.

**Figure 1.**
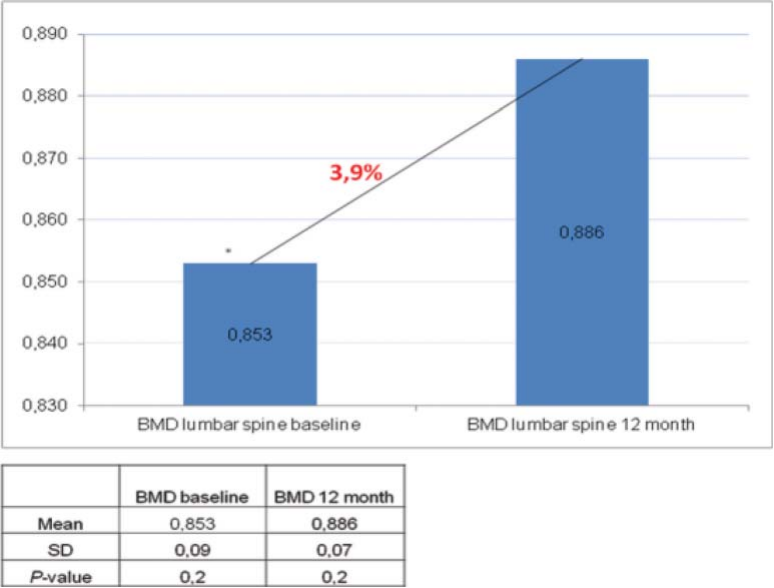
Effect of denosumab on BMD at the lumbar spine.

**Figure 2.**
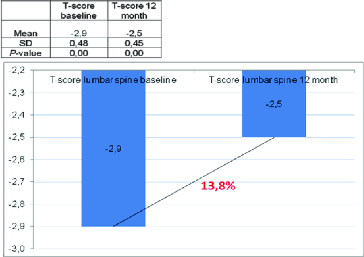
Change in T-score of lumbar spine.

**Figure 3.**
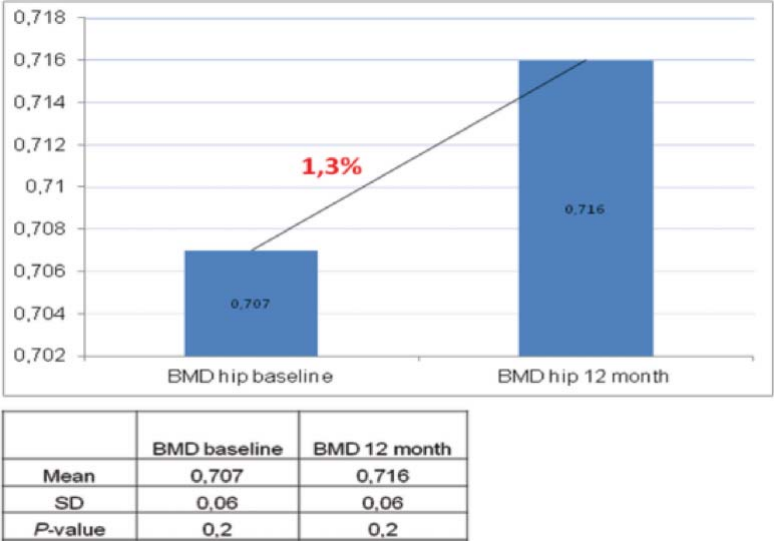
Effect of denosumab on BMD at the total hip.

**Figure 4.**
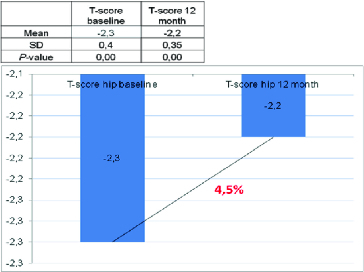
Change in T-score of total hip.

**Figure 5.**
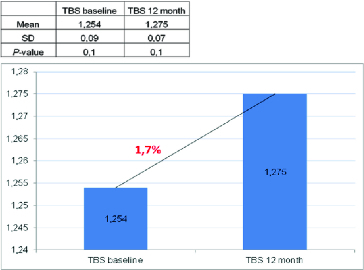
Effect of denosumab on TBS.

**Figure 6.**
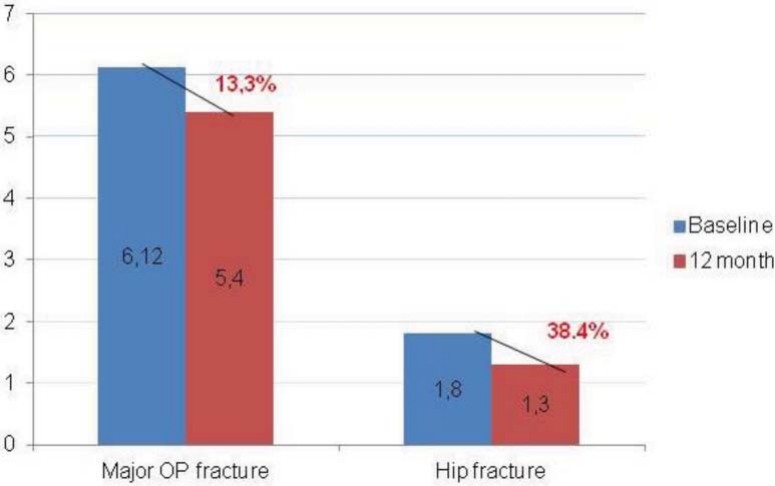
Fracture risk reduction after a 12-month treatment with denosumab.

Diagnosis Press and Taylor & Francis apologize for this error.

